# Study on Optimized Elman Neural Network Classification Algorithm Based on PLS and CA

**DOI:** 10.1155/2014/724317

**Published:** 2014-08-06

**Authors:** Weikuan Jia, Dean Zhao, Tian Shen, Yuyang Tang, Yuyan Zhao

**Affiliations:** ^1^School of Electrical and Information Engineering, Jiangsu University, Zhenjiang 212013, China; ^2^Changzhou College of Information Technology, Changzhou 213164, China

## Abstract

High-dimensional large sample data sets, between feature variables and between samples, may cause some correlative or repetitive factors, occupy lots of storage space, and consume much computing time. Using the Elman neural network to deal with them, too many inputs will influence the operating efficiency and recognition accuracy; too many simultaneous training samples, as well as being not able to get precise neural network model, also restrict the recognition accuracy. Aiming at these series of problems, we introduce the partial least squares (PLS) and cluster analysis (CA) into Elman neural network algorithm, by the PLS for dimension reduction which can eliminate the correlative and repetitive factors of the features. Using CA eliminates the correlative and repetitive factors of the sample. If some subclass becomes small sample, with high-dimensional feature and fewer numbers, PLS shows a unique advantage. Each subclass is regarded as one training sample to train the different precise neural network models. Then simulation samples are discriminated and classified into different subclasses, using the corresponding neural network to recognize it. An optimized Elman neural network classification algorithm based on PLS and CA (PLS-CA-Elman algorithm) is established. The new algorithm aims at improving the operating efficiency and recognition accuracy. By the case analysis, the new algorithm has unique superiority, worthy of further promotion.

## 1. Introduction

Neural network (NN) [1] is a nonlinear and adaptive information processing system based on the intelligent computation of the computer network simulating biological neural network, which processes and memorizes information by simulating cranial nerve and consists of large interconnect processing unit. Elman neural network [2] is a feedback neural network, which is optimized based on the research of backpropagation (BP) neural network [3] in 1990 by Elman; it is to add a connecting layer to the hidden layer of feedforward network as time delay operator so as to memorize and, therefore, make the system have the characteristic of time-varying and have stronger global stability. However, BP neural network has some inherent disadvantages [4], like easiness to fall into local minimum, the fixed learning rate, difficulty to determine the number of hidden layer neurons, and so on. Elman neural network certainly inherited these deficiencies. Even so, Elman neural network is proposed more than 20 years and is widely used in many fields [5–7]. Neural network provides great convenience to solve the complex problem for people, but it is not perfect for the nature of sample to be dealt with and the network's algorithm or structure. Such as the nature of sample, when we obtain the sample data, we always want to collect the feature information as much as possible; they can provide sufficient data support to solve the problem. However, a large number of features' information provide available information; at the same time, they also increase the difficulty for the neural network to deal with these data; the useful information is always buried in redundant data. Among the features, the samples may present some correlative or even repetitive information, so it will occupy a lot of storage space and consume much computing time. With too many feature inputs, this will harm the design of the network structure, prevent the convergence of neural network, and influence the training speed of neural network; too many feature inputs and repetitive sample training will make the training process time-consuming; repetitive sample also influence the training speed of neural network. These factors will influence the operating efficiency of neural network or even cause network paralysis, ultimately affecting the recognition accuracy of neural network.

Using the neural network deal with the complex sample, it is necessary to preprocess the original data. The high feature dimension of the sample adopts the theory of dimension reduction [8–10]; it analyzes and extracts useful variable features from the massive data, removes the influence of correlative or redundant factors, and reduces the dimension of the variable features as much as possible under the premise of not affecting the question solution; this is regarded as longitudinal dimension reduction. The purpose is to simplify the network structure and improve the convergence speed. The correlation of the samples, through the cluster analysis, clusters the similar samples into a subclass, refining the sample to eliminate individual outlier samples, and divides all samples into some subclasses. Each subclass is regarded as new sample to train neural network; the purpose is to get different more precise neural network model, thus improving recognition accuracy. So the cluster analysis can be regarded as sample horizontal dimension reduction. Integrating the dimension reduction and neural network organically is one of the hotspots of the neural network algorithm improvement at present and has got gratifying achievements. Integrating the principal component analysis (PCA) and neural network [11, 12], through PCA, reduces dimension and simplifies the procedure of neural network training. Integrating the factor analysis (FA) and neural network [13, 14], FA can successfully reduce inputs, with little information loss, and speed up the neural network computing. In addition, integrating the independent component analysis (ICA) and neural network [15, 16], through the ICA, eliminates noise effects and improves the network convergence speed and recognition accuracy.

Relatively speaking, these improvements are only for sample feature; that is, they cannot be taken into account for the relationship between individual samples. Aiming at the nature between samples, introduce the cluster analysis (CA) into the neural network algorithm. By cluster analysis, cluster the similar samples into one class and eliminate some outlier samples which will influence the network's recognition accuracy. Regard every sub-class as a new sample and train the neural network for each subclass to get a more precise model, which improves the recognition accuracy. So cluster analysis can also be considered as sample dimensionality reduction or horizontal dimensionality reduction, and now combining cluster analysis with neural network has been applied in some research [17, 18].

Integrating the longitudinal dimension reduction, cluster analysis, and neural network has also some relevant research applications. Lin et al. used neural network based on CA and PCA to carry on the blast furnace data test [19]. Ding combines FA, CA, and BP neural network and proposes the FA-CA-BP algorithm [20]. The above two algorithms do longitudinal dimension reduction without considering clustering analysis for the sample. Through the cluster analysis, divide all samples into different close subclasses; they may become small sample, with high-dimensional feature and few numbers. Using the traditional dimension reduction algorithm for small sample reduces dimension; it is difficult to achieve the ideal result. In order to prevent the problem of small sample caused by cluster, we introduce partial least squares (PLS) dimension reduction algorithm [21, 22] into neural network algorithm, which can fully take into account the level of correlation between feature variables and the dependent variables; can get the relatively ideal low-dimensional data. Using PLS method to extract the feature variables has achieved good application [23, 24]; optimized neural network based on PLS also has certain progress [25, 26].

Based on the above studies, in this paper, we introduce the PLS and CA into Elman neural network algorithm and establish an optimized classification Elman neural network based on PLS and CA; the new algorithm purpose is to improve operating efficiency and recognition accuracy. New algorithm by PLS longitudinal dimension reduction has three advantages: the first, it gets the low-dimensional data as the networks inputs, simplifies the network structure, and improves the network's operating efficiency; the second, it can fully take into account the level of correlation feature variables and the dependent variables and then get the ideal low-dimensional data with strong interpretation; and the third, it resolves the problem of the subclass becoming small sample by clustering. Through CA horizontal dimension reduction, comparing the correlation between the nature of the samples, the influence of the correlative factors and the noise of among samples is eliminated and the similar samples get gathered to close subclass; it can detail the samples, targeted training, and each subclass can get a precise neural network model. Then classify the simulation samples to different subclasses, with the corresponding subclass trained neural network model to to recognize the simulation sample; thus improving the recognition accuracy of neural network.

For illustrating the validity of algorithm, use traditional BP neural network, traditional Elman neural network, Elman neural network based on principal component analysis (PCA-Elman algorithm) [27], PLS-Elman algorithm, and CA-Elman algorithm, respectively, to test the case, from the aspects of training step and sum of square error and success rate of recognition, to contrast the test results. The new algorithm has unique superiority, worthy of further promotion.

## 2. The PLS-CA-Elman Neural Network Classification Algorithm

### 2.1. Basic Principle of Elman Neural Network

**Figure 1:**
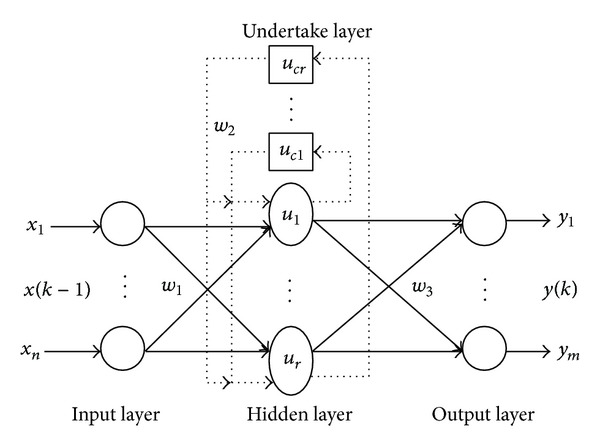
The topology structure of Elman neural network.

Elman neural network is a kind of feedback neural network; based on BP neural network hidden layer adds an undertake layer, as the delay operator, the purpose of memory, so that the network system has ability to adapt to the time-varying dynamic characteristics and has strong global stability.  Figure 1 shows Elman neural network structure.

The topology is generally divided into four layers: input layer, hidden layer, undertake layer, and output layer. Undertake layer is used to remember the output of hidden layer, which can be seen as a step delay operator. Based on BP network, the output of hidden associates with its input through the delay and storage of undertake layer. This way of association is sensitive to historical data, and internal feedback network can increase the ability of handing dynamic information. Remembering the internal state makes it have dynamic mapping function, which makes the system have the ability to adapt to time-varying characteristics.

Suppose with *n* input, *m* output, the number of hidden and undertake neurons are *r*, the weight of input layer to hidden layer is *w*_1_, the weight of undertake layer to hidden layer is *w*_2_, the weight of hidden layer to output layer is *w*_3_; *u*(*k* − 1) is the input of neural network, *x*(*k*) is the output of hidden layer, *x*_*c*_(*k*) is the output of undertake layer, and *y*(*k*) is the output of neural network; then

(1) $$\begin{array}{c} x\left(k\right)=f\left(w_{2}x_{c}\left(k\right)+w_{1}\left(u\left(k-1\right)\right)\right), \end{array}$$

where

(2) $$\begin{array}{c} x_{c}\left(k\right)=x\left(k-1\right); \end{array}$$

*f* is the hidden layer transfer function, which is commonly used in S-type function; that is,

(3) $$\begin{array}{c} f\left(x\right)={\left(1+e^{-x}\right)}^{-1}; \end{array}$$

*g* is the transfer function of output layer, which is often a linear function; that is,

(4) $$\begin{array}{c} y\left(k\right)=g\left(w_{3}x\left(k\right)\right). \end{array}$$

Elman neural network uses BP algorithm to revise weights; the error of network is

(5) $$\begin{array}{c} E=\overset{m}{\underset{k=1}{\sum }}{\left(t_{k}-y_{k}\right)}^{2}, \end{array}$$

where *t*_*k*_ is the output vector of object.

### 2.2. PLS Longitudinal Dimension Reduction

Partial least squares is the characteristic development of ordinary least squares; the basic idea is that the characteristic variable matrix *X* is compressed, at the same time, giving consideration to the correlation of the dependent variable matrix *Y*. Suppose that there are *n* characteristic variables, *x*_1_, *x*_2_,…, *x*_*n*_, *p* dependent variables, *y*_1_, *y*_2_,…, *y*_*p*_; after preprocessing, matrix *X* is decomposed into

(6) $$\begin{array}{c} X=TP^{T}+E, \end{array}$$

where *T* is the score matrix, *P* is the load matrix, and *E* is the residual error matrix. Matrix multiplication *TP* ^*T*^ can be expressed as the sum products of score vector *t*_*i*_ (the *i*th column of matrix *T*) and load vector *p*_*i*_ (the *i*th column of matrix *P*); then the above formula can be written as

(7) $$\begin{array}{c} X=\overset{n}{\underset{i=1}{\sum }}t_{i}p_{i}^{T}+E,\;i=1,2,\ldots ,n. \end{array}$$

Similarly, matrix *Y* is decomposed into

(8) $$\begin{array}{c} Y=UQ^{T}+F, \end{array}$$

where *U* is the score matrix, *Q* is the load matrix, and *F* is the residual error matrix. Matrix multiplication *UQ* ^*T*^ can be expressed as the sum products of score vector *u*_*j*_ (the *j*th column of matrix *U*) and load vector *q*_*j*_ (the *j*th column of matrix *Q*); then the above formula can be written as

(9) $$\begin{array}{c} Y=\overset{p}{\underset{j=1}{\sum }}u_{j}q_{j}^{T}+F,\;j=1,2,\ldots ,r. \end{array}$$

PLS analysis is separately extracted from the scores  *t* and *u* from corresponding *X* and *Y*; they are the linear combination of characteristic variables and dependent variables. And both scores satisfied the maximum load of variation information of characteristic variables and dependent variables; the covariance between them is the largest. Establish the regression equation

(10) $$\begin{array}{c} u_{j}=b_{k}t_{i}, \end{array}$$

where *b*_*k*_ is regression coefficient; the formula can be expressed in matrix form as

(11) $$\begin{array}{c} Y=BX, \end{array}$$

where *B* is coefficient matrix:

(12) $$\begin{array}{c} B=W{\left(P^{T}W\right)}^{-1}Q^{T}, \end{array}$$

where *W* is the weight matrix.

PLS aims that each dimension do iterative calculation using each other's information, each iteration continuously according to residual information of *X*, *Y* to adjust *t*_*i*_, *u*_*j*_ for the second round extracted, until the residual matrix element of absolute value approximates to zero; if the precision satisfied the requirements, the algorithm stops. In the iteration process, *t*_*i*_, *u*_*j*_ can maximize the expression of variance of *X* and *Y* at the same time.

PLS regression does not need to use all the components to establish the regression equation; it only needs selecting the front *l* components (0 ≤ *l* ≤ *n*) and then can get better regression equation. Generally use *K*-fold cross-validation method to calculate prediction residual sum of squares to determine the number of components extracted, reaching the purpose of dimension reduction.

### 2.3. CA Horizontal Dimension Reduction

Cluster analysis is one method to study “birds of a feather flock together”; it is to classify the samples according to the different relations of their characteristics. The different relations have two kinds of representations: one is to regard each sample as a point in the *m*-dimensional space and then to define some distance between points and points in the *m*-dimensional coordinate axis; the other is some kind of similarity coefficient.

Here we use hierarchical cluster analysis (HCA). HCA is a kind of the most widespread methods in the cluster analysis. The basic idea is that, when there are *n* samples, each sample is one class, firstly, calculate *C*_*n*_^2^ similar measures and combine the two smallest measure samples into a class of two elements and secondly calculate the distance between this class and other *n*-2 samples according to some HCA method. In the process of combining categories, each step must make the combined classes to keep measure smallest in the system and reduce one class every time, until all the samples are classified as one class. In order to overcome the related influence between variables, the measure uses Mahalanobis distance and uses the commonly used unweighted pair-group method with arithmetic means (UPGMA) to cluster the classes.

Suppose that samples *X*, *d*_*ij*_ express the Mahalanobis distance between the samples *x*_*i*_ and *x*_*j*_:

(13) $$\begin{array}{c} d_{ij}={\left(x_{i}-x_{j}\right)}^{\prime }V^{-1}\left(x_{i}-x_{j}\right),\;i,j=1,2,\ldots ,n, \end{array}$$

where *x*_*i*_ represents the *i*th vector and *V* ^−1^ is the inverse matrix of the sample covariance matrix; the covariance matrix of the sample is

(14) $$\begin{array}{c} V_{ij}=\frac{\sum_{k=1}^{n}\left(x_{ik}-\bar{x_{i}}\right)\left(x_{jk}-\bar{x_{j}}\right)}{n-1}. \end{array}$$

UPGMA calculates the distance between samples, gains the distance matrix, finds out the smallest element from the distance matrix, and combines the two classes into a new class. Then it calculates the class average distance between the two classes, using *D*_*pq*_ to represent

(15) $$\begin{array}{c} D_{pq}=\frac{1}{n_{p}n_{q}}\sum_{i\subset G_{p},j\subset G_{q}}d_{ij}^{2}, \end{array}$$

where *G*_*r*_ = {*G*_*p*_, *G*_*q*_}, *n*_*r*_ = *n*_*p*_ + *n*_*q*_; *n* represents the number of the samples in the classes. The distance between the *G*_*k*_ and *G*_*r*_ is

(16) $$\begin{array}{c} D_{kr}=\frac{n_{p}}{n_{r}}D_{kp}^{2}+\frac{n_{q}}{n_{r}}D_{kq}. \end{array}$$

A new distance matrix can be gained. Then combine the two classes which have smallest distance, by parity of reasoning, until combining to one class. We should control the number of the subclasses by *λ* cutoff value. According to the actual problems, generally we cluster the data after dimension reduction into 3–6 categories. Each subclass is regarded as a new sample.

### 2.4. PLS-CA-Elman Neural Network Algorithm

#### 2.4.1. The Thought of New Algorithm

Elman neural network is kind of optimizing BP neural network, so it inherits the characteristics of BP, but BP has some defects; for example, it is easy to fall into local minimum, the fixed learning rate, the uncertain number of hidden layer neurons, and if encountering small sample this is more challenging. In order to improve operating efficiency and recognition accuracy of neural networks, we introduce PLS and CA into Elman neural networks algorithm. Using PLS longitudinal dimension reduction, it can fully take into account the level of correlation between feature variables and the dependent variables and can solve the small sample problems caused by cluster analysis. Through cluster analysis, similar samples are classified as one close subclass and easy to train more precise neural network model. We establish an optimized Elman neural network classification algorithm based on partial least squares and cluster analysis (PLS-CA-Elman algorithm); the thought of new algorithm has a variety of structures. 

**Figure 2:**
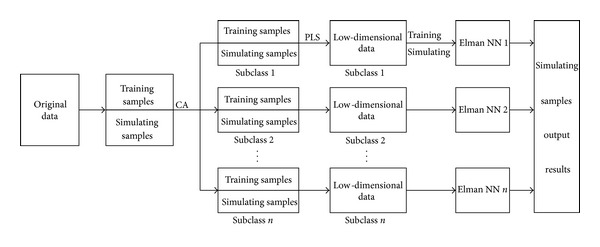
The algorithm flow chart of Thought 1.

*Thought 1*. First, according to the need of the problem, factitiously, divide all samples into training sample and simulation sample. Second, carrying out cluster analysis on all samples, we can get some different close subclasses; each subclass may contain training sample and simulation sample. Some subclass may become small sample, so do longitudinal dimension reduction by PLS. Third, for each class, using training sample to train Elman neural network, getting a precise neural network model is ensured; using corresponding trained model recognize simulation samples in the subclass, which will give accurate recognition results.  Figure 2 is the flow chart of Thought 1.

 
*Advantage*: to reduce the dimension of each cub-class by PLS, and obtained the low-dimensional data with stronger explanatory.
 
*Disadvantage*: getting the subclass may contain few training samples and many simulation samples; the neural network cannot be trained.

**Figure 3:**
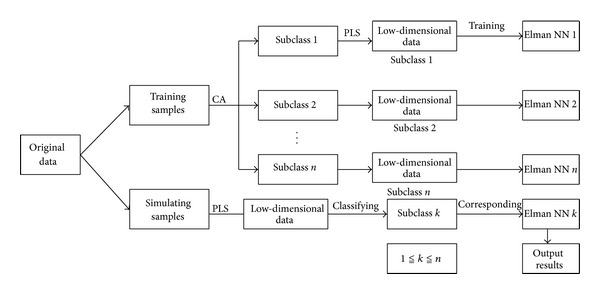
The algorithm flow chart of Thought 2.

*Thought 2*. First, divide the original data into training sample and simulation sample. Second, carrying out cluster analysis on training samples, we can get some different close subclasses; each subclass is regarded as new sample, which somehow may be small sample. Third, each subclass and simulation sample used PLS method for longitudinal dimension reduction, using low-dimensional data training neural network for each subclass. Finally, the simulation samples are discriminated one by one and classified into different subclasses; then using the corresponding trained neural network to to recognize the simulation samples. One can obtain more accurate recognition results.  Figure 3 is the flow chart of Thought 2.

 
*Advantage*: to ensure each class contains enough samples for training neural network.
 
*Disadvantage*: each sub-class and simulation sample for dimension reduction may be out of step, to extract the feature dimension is different, lead to simulation sample cannot be discriminated and recognized; the simulation samples classify one by one, will consume more computing time.

**Figure 4:**
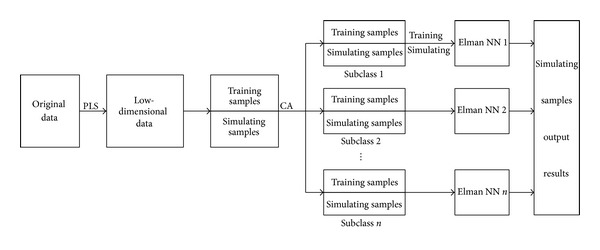
The algorithm flow chart of Thought 3.

*Thought 3*. First, using PLS dimension reduction algorithm on the original data, to get low-dimensional data, factitiously, conventions divided all samples into training sample and simulation sample. Second, carrying out cluster analysis on all samples, we can get some different close subclasses, which may contain training sample and simulation sample. Third, for each subclass, using training sample to train Elman neural network, getting a precise neural network model is ensured. Finally, using corresponding model recognize simulation samples in the sub-class, will get accurate recognition results.  Figure 4 is the flow chart of Thought 3.

 
*Advantage*: once clustered, divide all samples into attribution.
 
*Disadvantage*: getting the subclass may contain few training samples and many simulation samples; the neural network cannot train successfully.

**Figure 5:**
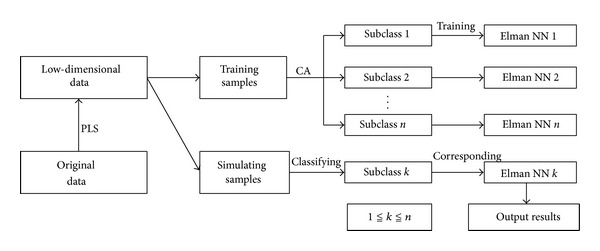
The algorithm flow chart of Thought 4.

*Thought 4*. First, using PLS dimension reduction on the original data, to get low-dimensional data as new sample, factitiously, conventions divided all samples into training sample and simulation sample. Second, carrying out cluster analysis on training samples, we can get some different close subclasses; then training the Elman neural network, we can get the precise model. Third, the simulation samples are discriminated one by one and classified into different subclasses; then using the corresponding trained model to to recognize the simulation samples, one can obtain more accurate recognition results.  Figure 5 is the flow chart of Thought 4.

 
*Advantage*: able to ensure that each class contains enough samples for neural network training.
 
*Disadvantage*: the simulation samples are classified one by one, which will consume more computing time.

#### 2.4.2. The Constructs of New Algorithm

**Figure 6:**
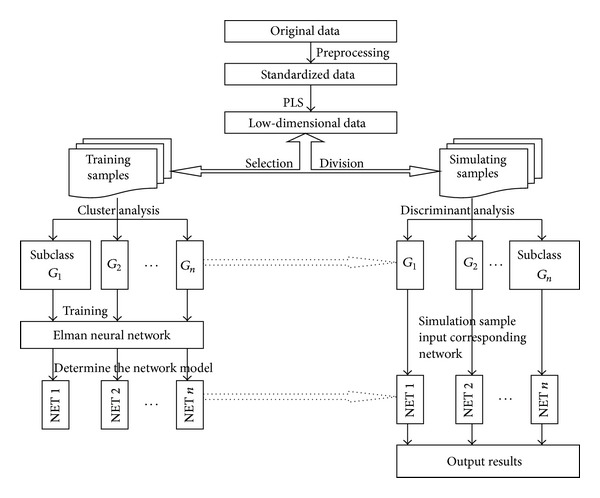
The flow chart of optimized Elman neural network classification algorithm based on PLS and CA.

In this paper, we adopt the fourth algorithm thought; establishing PLS-CA-Elman optimal algorithm, the new algorithm is described as follows. First, using PLS longitudinal dimension reduction algorithm on the original data to get low-dimensional data, we divide all samples into training sample set and simulation sample set. Second, we carry out cluster analysis on training samples, according to the need of the actual problem, to determine the number of subclasses. Third, each subclass is regarded as one new sample to train Elman neural network, which will give the precise neural network model, so each subclass corresponds one trained model. Finally, respectively, we calculate the distance from each simulation sample to each subclass' center and, according to the distance, discriminate simulation samples into different subclasses. Using the subclass corresponding Elman neural network model to recognize simulation samples, we get the simulation result.  Figure 6 shows the flow chart of the new algorithm.

#### 2.4.3. Set the Parameters of New Algorithm

Preset the basic parameters of optimization algorithms. 

(*1) Preprocess the Original Data Sets*. The original data preprocess: the purpose is to eliminate the different indicator distributions, the noncomparability which is caused by the differentia of the data, and to guarantee the quality from the data source by standardized processing to the original data. Processed data are consistent with *N*(0,1) distribution; standardization conversion formula is

(17) $$\begin{array}{c} x_{ij}^{\prime }=\frac{\left(x_{ij}-\bar{x_{j}}\right)}{\sqrt{\left(1/n\right)\sum_{i=1}^{n}{\left(x_{ij}-\bar{x_{j}}\right)}^{2}}}. \end{array}$$

(*2) Parameters of PLS*. PLS feature dimension reduction using *K*-fold cross-validation (*K*-CV) method: calculate prediction residual sum of squares. This method can effectively avoid overlearning or owe-learning, getting the result with more persuasive, obtaining the low-dimensional data has more interpretation. 

(*3) Parameters of CA*. Carrying out cluster analysis horizontal dimension reduction algorithm: here we use hierarchical cluster analysis (HCA). HCA is a kind of the most widespread methods in the cluster analysis. The similarity measure uses mahalanobis distance in order to overcome the correlative influence between variables, and distance calculationcriterion uses the commonly used UPGMA to cluster the classes. We should control the number of the categories by *λ* cutoff value; according to the actual problems, generally we cluster the data after dimension reduction into 3–6 classes. 

(*4) The Parameters of Elman Neural Network*. Set the parameters of Elman neural network algorithms: the undertake layer and hidden layer have the same number of neurons, using Gao Daqi's empirical formula to determine the number of hidden neurons [28]

(18) $$\begin{array}{c} s={\left(0.43nm+0.12m^{2}+2.54n+0.77m+0.35\right)}^{1/2}+0.51. \end{array}$$

In the formula, *s*, *n*, and *m*, respectively, express the number of neurons of hidden layer, input layer, and output layer. The neural network training uses trainlm (LM) algorithm, which is the combination of gradient descent and quasi-newton algorithm; its advantage is giving full play to the gradient descent algorithm which has convergence speed at the beginning of training steps and quasi-newton algorithm near the extremum can quickly produce an ideal search direction. Connection weights and threshold learning use the learngdm algorithm.

#### 2.4.4. The Basic Steps of New Algorithm

The PLS-CA-Elman optimal algorithm basis steps are described as follows.

**Step 1 .**

Normalize the original data using the formula (17) and get the characteristic variables matrix *X* and dependent variable matrix *Y*.

**Step 2 .**

Respectively, extract the first pair components *t*_1_, *u*_1_ from *X*, *Y* and make up to the maximum correlation.

**Step 3 .**

Respectively, establish the regression equation of *X* on *t*_1_ and *Y* on *u*_1_.

**Step 4 .**

To judge the absolute value of the residual matrix elements close to zero, if yes, turn to Step 6, or else turn to Step 5.

**Step 5 .**

Using residual error matrix *E* and *F* instead of *X* and *Y*, turn to Step 2.

**Step 6 .**

With *K*-CV method, calculate residual sum of squares and determine the number of components extracted.

**Step 7 .**

From the perspective of information feature compression, get the compression matrices *X* and *Y*, as new samples.

**Step 8 .**

Divide the new samples into two as training sample and simulation sample according to the need of the problem.

**Step 9 .**

Using formula (13) calculate the Mahalanobis distance between the training samples, gaining a distance matrix *D*.

**Step 10 .**

Combine the two categories that correspond the smallest element of *D* as a new category.

**Step 11 .**

By formula (15), calculate average distance between the center of the new class and other classes and get a new distance matrix *D*1, according to the actual problems, to determine whether meet the requirements number of clustering class, if yes, turn to Step 12, or else turn to Step 9.

**Step 12 .**

Each class is regarded as a new small sample, as training sample.

**Step 13 .**

By formula (13), calculate the distance between the every simulation sample and each class center and classify the simulation samples into the class.

**Step 14 .**

According to the feature dimension determined by Step 6 and the output of actual problem, use formula (18) to determine the number of hidden layer neurons.

**Step 15 .**

Use the new sample obtained by Step 12 and adopt LM algorithm to train the neural network.

**Step 16 .**

Adopt learngdm algorithm to determine the connect weights and threshold of the neural network.

**Step 17 .**

For each class corresponding a precise Elman neural network model, use the trained network to test the simulation sample and count the test results.

Processing the original data using PLS algorithm, for longitudinal dimension reduction at the premise of taking into account the level of correlation feature variables and the dependent variables, the main purpose is to reduce the input of network, in order to simplify the network structure, improve the training speed, and save the operation time, and also it can effectively solve the problem of small sample dimension reduction. The main purpose of samples clustering is to eliminate the correlation between samples; the similar samples cluster into a subclass in order to improve the recognition accuracy of the neural network.

In order to better verify the validity of the new algorithm, according to the above steps, we establish the Elman algorithm based on PLS (PLS- Elman), CA-Elman algorithm.

## 3. Experiment

The classification system, in essence, can be seen as an input-output system; the transformation relations include the data fitting, the fuzzy transformation, and the logic reasoning; these can all be represented by the neural network.

In order to better verify the superiority of the new algorithm, respectively, using BP neural network, Elman neural network, PCA-Elman algorithm, PLS-Elman algorithm, CA-Elman algorithm, and PLS-CA-Elman algorithm to test the case, compare the network performance and recognition results. The test results to measure the advantages and disadvantages of the algorithm form the following four aspects: recognition accuracy rate, training steps, run time, and the error sum of squares (the error square sum is the square sum of the difference between the predicted value and actual value). Because each clustering subclass needs neural network training, the training steps are not convenient to statistics, and the main purpose is to train more precise neural network model to improve the recognition accuracy, so the training steps of the CA-Elman algorithm and PLS-CA-Elman algorithm have no record.

In order to test the reliability of the new algorithm, we use two data sets for testing. One data set is actual production data; we choose set of wheat blossom midge in Guanzhong area [29]; another is UCI standard data sets; here we choose the ionosphere data subset of radar [30].

### 3.1. Test 1

We have known data, using the weather factors, to forecast the occurrence degree of the wheat blossom midge in Guanzhong area. Here we choose the data of 60 samples from 1941 to 2000 as the study object, with 14 feature variables and 1 dependent variable.

The tests choose the 45 samples from 1941 to 1985 as the training samples; use the 15 samples from 1986 to 2000 as the simulation samples. Utilizing the MATLAB toolbox, respectively, to test the traditional BP and Elman neural network algorithm, to train them by training sample, and then using the trained Elman neural network to recognize the simulation samples; the results are listed in Table 1.

Test by PCA-Elman algorithm, through PCA, can extract 6 principal components; the variance total contribution ratio reaches up to 80%, the dimension of the original data from 14 is reduced to 6, namely, the inputs is reduced. Using low-dimensional training sample to train the Elman neural network, and then using the trained Elman neural network to recognize the simulation samples; the results are listed in Table 1.

Test by PLS-Elman algorithm, through PLS, also extracts 6 principal components; the absolute value of the residual matrix elements is close to zero, the dimension of the original data reduces from 14 to 6, the dimension of the original data from 14 is reduced to 6, namely, the inputs is reduced. Using low-dimensional training sample to train the Elman neural network, and then using the trained Elman neural network to recognize the simulation samples; the results are listed in Table 1.

**Figure 7:**
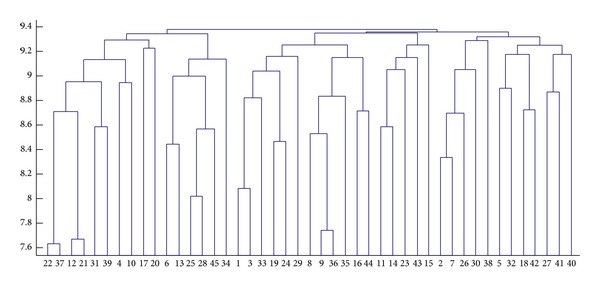
The graph of hierarchical cluster for original training samples.

For test by CA-Elman algorithm, by CA, the 45 training samples cluster into 3 subclasses; each subclass is regarded as a new training sample to train the Elman neural network, so we can get 3 Elman neural networks.  Figure 7 is the hierarchical cluster analysis graph of training samples, *λ* = 9.36; the training sample is divided into three subclasses. The simulation sample is classified to different classes by discriminant analysis, using corresponding trained Elman neural network to recognize simulation samples; the results are listed in Table 1.

**Figure 8:**
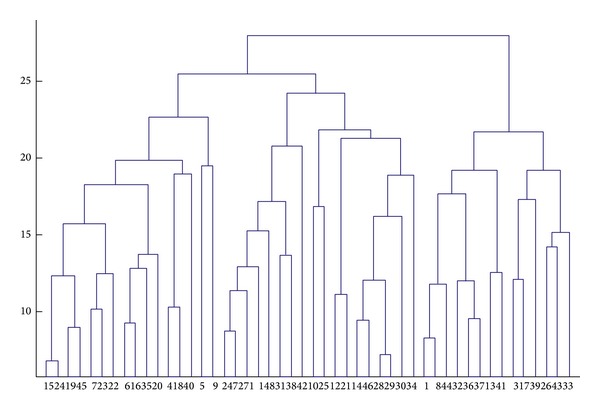
The graph of hierarchical cluster for low-dimensional training samples.

Test by PLS-CA-Elman algorithm, through PLS also extract 6 principal components, the dimension of the original data form 14 reduce to 6, through CA the 45 training samples cluster into 3 classes, ach class as a new training sample training the Elman neural network.  Figure 8 is the hierarchical cluster analysis graph of low-dimensional training samples, *λ* = 25, the training sample is divided into three sub-classes. The simulation sample classify to different class by discriminant analysis, using corresponding trained Elman neural network recognize simulation samples, the results are listed in Table 1.

### 3.2. Test 2

**Figure 9:**
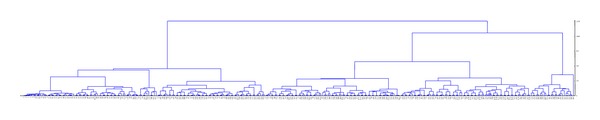
The graph of hierarchical cluster for original data of radar training samples.

We further test using UCI data set to simulate the ionosphere data subset of radar set; this has 351 samples; there are 34 features, which are used to predict the quality of the radar, with 34 inputs and 1 output. We selected the front 300 samples as training samples and the remaining 51 samples as the simulation samples.  Figure 9 is the hierarchical cluster analysis graph of training samples, *λ* = 810; the training sample is divided into three subclasses.

**Figure 10:**
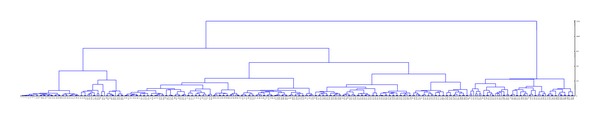
The graph of hierarchical cluster for low-dimensional data of radar training samples.

For PLS method extracted 16 principal components from 34 features, the dimension of the original data form reduced from 34 to 16.  Figure 10 is the hierarchical cluster analysis graph of low-dimensional training samples, *λ* = 600; the training sample is divided into three subclasses.

The simulation results of ionosphere radar data set are listed in Table 2.

### 3.3. Results

Tables 1 and 2 show that the results of the Elman neural network from three indicator aspects are better than BP neural network; they illustrate that the classification ability of Elman is better than BP neural network.

Comparing the classification results of PCA-Elman algorithm, PLS-Elman algorithm, and the Elman algorithm, the recognition accuracy rate is not reduced, but the step of the convergence is reduced; the sum square error also has been reduced; it illustrates that although the data lose some information by dimension reduction, this does not influence the recognition results.

Comparing the classification results of PLS-Elman algorithm with PCA-Elman algorithm, three indicators are dominant; it illustrates that the effect of feature extraction PLS is better than PCA, By PLS algorithm to obtain the low-dimensional data has more meaningful.

From the run time of each algorithm, comparing the optimized neural network algorithm by dimension reduction with traditional Elman neural network, no much different run time is consumed, which shows that the consume time of dimensionality reduction can to counteract the running time of the traditional neural network training, which shows that the consume time of dimensionality reduction can to counteract the running time of the traditional neural network training, but the optimized neural network algorithm by cluster analysis is higher than other algorithms obviously; it shows that through cluster analysis the computation complexity is increased, which will consume some running time. The PLS-CA-Elman algorithm consumes the longest running time.

Comparing the CA-Elman algorithm with aforementioned three algorithms, the recognition accuracy rate is sharply improved, sum square error also has been sharply reduced, and it illustrates that the recognition accuracy has sharply improved. PLS-CA-Elman algorithm has increased complexity in the operation, but we can achieve satisfactory results on recognition accuracy, network design, and the convergence speed.

## 4. Discussion

Comparing Elman neural network with BP neural network, it is to add a connecting layer to the hidden layer of feedforward network as time delay operator so as to memorize and it is used to form local feedback. Elman neural network most used to spatio-temporal pattern recognition, its result is better than BP neural network.

When faced with the complex large sample data, by longitudinal dimension reduction, dealing with original data has certain information loss; however, it can reduce the data redundancy, also it removes the influence of the correlative and repetitive data, under the premise of uninfluenced to solve the problem to reduce the data feature dimension, and reduces the network inputs, simply the network structure, thus improving the operating efficiency. The test results show that compared to the longitudinal dimension reduction optimized Elman algorithm with traditional Elman algorithm, the recognition accuracy rate is not reduced, but the step of the convergence is reduced, the convergence speed is increased, the operation time is saved, and the sum square error is slightly reduced; it illustrates that through the longitudinal dimension reduction optimized Elman neural network algorithm, the operating efficiency has improved. About the neural network algorithm based longitudinal dimension reduction, PLS-Elman algorithm of every indicator is better than PCA-Elman algorithm, it illustrates that the obtained low-dimensional data PLS algorithm is more significant than PCA algorithm, with better dimension reduction effect, which is more conductive to solve the problem.

Through CA for horizontal dimension reduction to training sample, the similar samples are classified into a subclass. All subclasses achieved “within the class homogeneity and heterogeneity between classes.” Each subclass trains one precise Elman neural network model. Then the simulation samples are analyzed by discriminant analysis to classify one by one, using the subclass corresponding network to recognize the simulation samples. The test results show that CA-Elman algorithm's recognition accuracy rate has been sharply improved and the sum square error has been sharply reduced; it illustrates that through CA optimized Elman neural network algorithm, it has improved the recognition accuracy.

About the selection of cluster algorithm, this paper uses hierarchical cluster analysis, for example, to verify the feasibility of cluster algorithm combined with neural network. Aiming at the different data sets, there may be the better clustering method, like K-means cluster, self-organizing map cluster, and other optimized cluster methods, and so forth. We will focus on the operating efficiency and recognition precision in the further work, so the requirements of cluster algorithm are higher than before. Next study, we will compare effect of different cluster algorithms combined with neural network to find the best classification algorithm.

In this paper, based on the Elman neural network, combined with the theory of partial least squares and cluster analysis, we establish an optimized Elman classification algorithm based on PLS and CA (PLS-CA-Elman algorithm). New algorithm by PLS longitudinal dimension reduction can eliminate the influence of the correlative factors between features. PLS algorithm can make full use of the data information in the system; the feature variables are decomposed and filtered, and extracted comprehensive variables have strong explanatory, meanwhile taking into account the degree of correlation between the feature variables and the dependent variables. Using the low-dimensional data training the neural network to reduce the inputs is beneficial to design the network structure, speed up the network convergence, and improve the learning efficiency of neural network. New algorithm by CA horizontal dimension reduction can eliminate the influence of the correlative factors between samples. The similar samples classify into one subclass, can get more precise neural network, and improve the recognition accuracy of the neural network. Elman neural network is one feedback neural network; it adds a connecting layer to the hidden layer of feedforward network as time delay operator so as to memorize and, therefore, makes the system have the characteristic of time-varying and have stronger global stability.

The PLS-CA-Elman algorithm is organic combination of the advantages of three algorithms, although increase operation complexity and sacrifice some running time, but we can achieve satisfactory results on recognition accuracy, network design, and the convergence speed. The most important of new algorithm is that its recognition precision has been improved greatly, and this is the final purpose of classification algorithm.

In this paper, use two data sets to test the effectiveness of new algorithm and achieve good results. In the following study, we will choose the bigger and more complex data sets to test new algorithm and to ensure obtaining the classification algorithm with better ability of generalization and classification.

## Figures and Tables

**Table 1:** The comparison of the performance of every algorithm for Test 1.

NN algorithm	Accuracy, %	Training steps	Run time, s	SSE
BP algorithm	73.33	1383	11.2	12.3659
Elman algorithm	80.00	956	9.6	9.7768
PCA-Elman algorithm	80.00	749	9.4	8.6293
PLS-Elman algorithm	86.67	532	9.6	6.2079
CA-Elman algorithm	100.00	—	15.1	2.0013
PLS-CA-Elman algorithm	100.00	—	18.3	1.2641

**Table 2:** The comparison of the performance of every algorithm for Test 2.

NN algorithm	Accuracy, %	Training steps	Run time, s	SSE
BP algorithm	72.55	1179	10.5	21.7214
Elman algorithm	82.35	1035	10.4	10.5267
PCA-Elman algorithm	84.31	878	9.7	9.0165
PLS-Elman algorithm	88.24	391	9.3	7.9570
CA-Elman algorithm	98.03	—	16.8	4.6673
PLS-CA-Elman algorithm	98.03	—	19.2	2.8926
